# Harvesting mouse suprachiasmatic nucleus by vibrating microtome for diurnal transcriptome analysis

**DOI:** 10.1016/j.xpro.2023.102618

**Published:** 2023-09-26

**Authors:** Akanksha Bafna, Petrina Lau, Gareth Banks, Patrick M. Nolan

**Affiliations:** 1 Medical Research Council, Harwell Science Campus, Oxfordshire, UK; 2 Wolfson Institute for Biomedical Research, University College London, London, UK

**Keywords:** Genetics, Model Organisms, Molecular Biology, Neuroscience

## Abstract

The mammalian suprachiasmatic nucleus (SCN) is the principal circadian clock that synchronizes daily behavioral and physiological responses in response to environmental cues. Here, we present a protocol for harvesting mouse SCN by vibrating microtome for diurnal transcriptome analysis. We describe steps for mouse entrainment, isolation of the SCN, tissue preparation, slicing with a vibratome, and handling of the harvested SCN for RNA extraction. This protocol can also be used for harvesting other mammalian brain regions for genomic studies.

## Highlights

• Extraction of highly viable SCN tissue from the adult mouse brain using vibratome
• Precisely timed tissue harvest to detect circadian transcriptional rhythms
• Full description of mouse entrainment, SCN collection, and rhythmicity analysis
• A reference protocol for harvesting mammalian brain regions for genomic studies

## Abstract (editor highlights)

Publisher’s note: Undertaking any experimental protocol requires adherence to local institutional guidelines for laboratory safety and ethics.

## Graphical abstract

**Figure:**
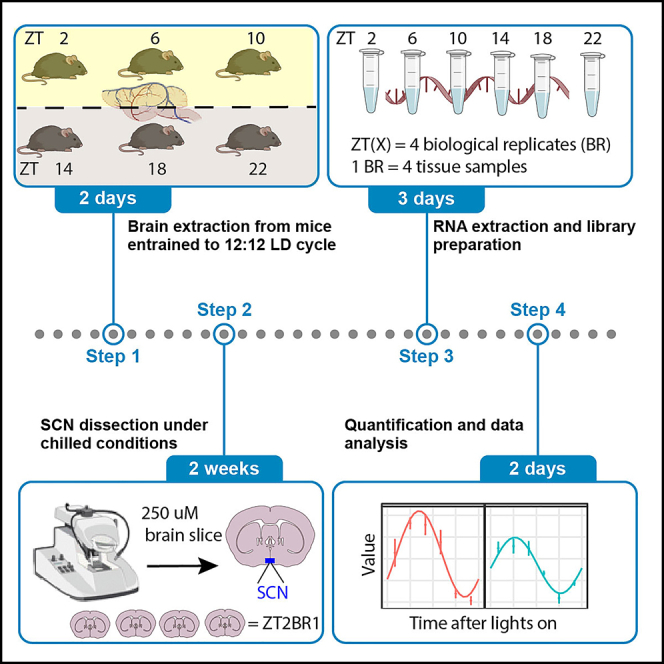

## Before you begin

The protocol below describes the specific steps for harvesting and extracting RNA from the SCN. However, this protocol can be applied to any brain region of interest when followed judiciously.

### Institutional permissions (if applicable)

All animal studies were performed under the guidance issued by Medical Research Council in Responsibility in the Use of Animals for Medical Research (July 1993) and Home Office Project Licence 19/0004. C57BL/6J were maintained and provided in-house by MRC Harwell.

### Mouse entrainment

**Timing: 2 weeks**

The animals are entrained under 12:12 h (hr) light-dark conditions. The number of mice to be used in the study should be carefully planned. For daily transcriptome profiling, we recommend harvesting mouse tissue(s) in 4-h interval over 24-h period. We used 3–4 biological replicates per time-point, where each biological replicate was a pool of four individual tissues (SCN). In total, we entrained and used ca. 96 animals, composed of equal number of males and females for this study.

**Figure 1:**
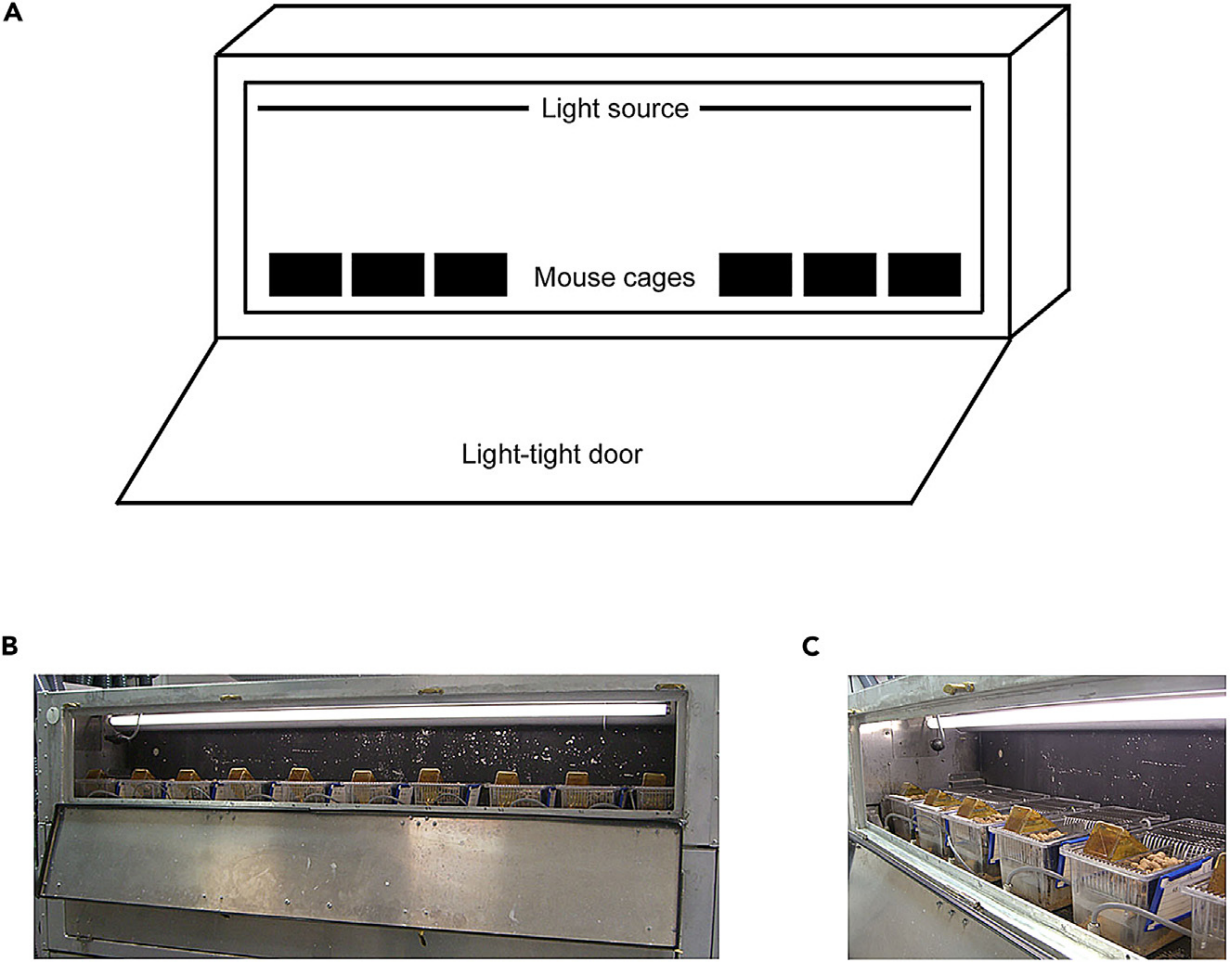
Mice in individual ventilated cages in light-controlled circadian chambers (A) Outline of circadian chambers. Mouse cages are placed inside light tight chambers with a light source above them. (B) Image of circadian chamber in use. (C) Detail of mouse cages inside circadian chamber.
  b. Use a staggered light schedule when using multiple light-controlled circadian chambers for the ease of harvesting tissue(s) at distinct times during the day.

***Note:*** For collecting tissues at night times, animals can be placed in cages inside a LCC programmed for lights on at 6 pm (ZT0) and lights off at 6am (ZT12). In this instance, for example, tissue(s) can be harvested from animals entrained at 8 am corresponding to ZT14.

1. Singly house adult mice (8–12 weeks old) in individually ventilated cages fitted with running wheels, under 12:12 h (hr) light-dark (LD) conditions (∼150 lux) with food and water available ad libitum.^1^ Alternatively, if space and time are limiting, mice strains (such as C57BL6/J) that are known to entrain efficiently to an exposed LD cycle can be group housed in conventional cages inside a light-controlled circadian chamber (LCC).
  a. Place 8–10 cages in a specialized light-controlled circadian chamber^2^ (air flow, temperature and humidity maintained according to institutional recommendations) programmed for lights on at 7 am (Zeitgeber time; ZT = 0) and lights off at 7 pm (ZT = 12) as shown in Figure 1.

2. Entrain mice to the 12:12 h light-dark schedule for 8–10 days. Change cage bedding on day seven if required.

**CRITICAL:** While the animals are under a light-dark schedule, minimize noise and cage disturbance as much as possible while maintaining animal welfare. It should be noted that light during lights-off would have a particularly detrimental effect on entrainment/experiment.

### Circadian behavior analysis (optional)

**Timing: 1 h**

**Figure 2:**
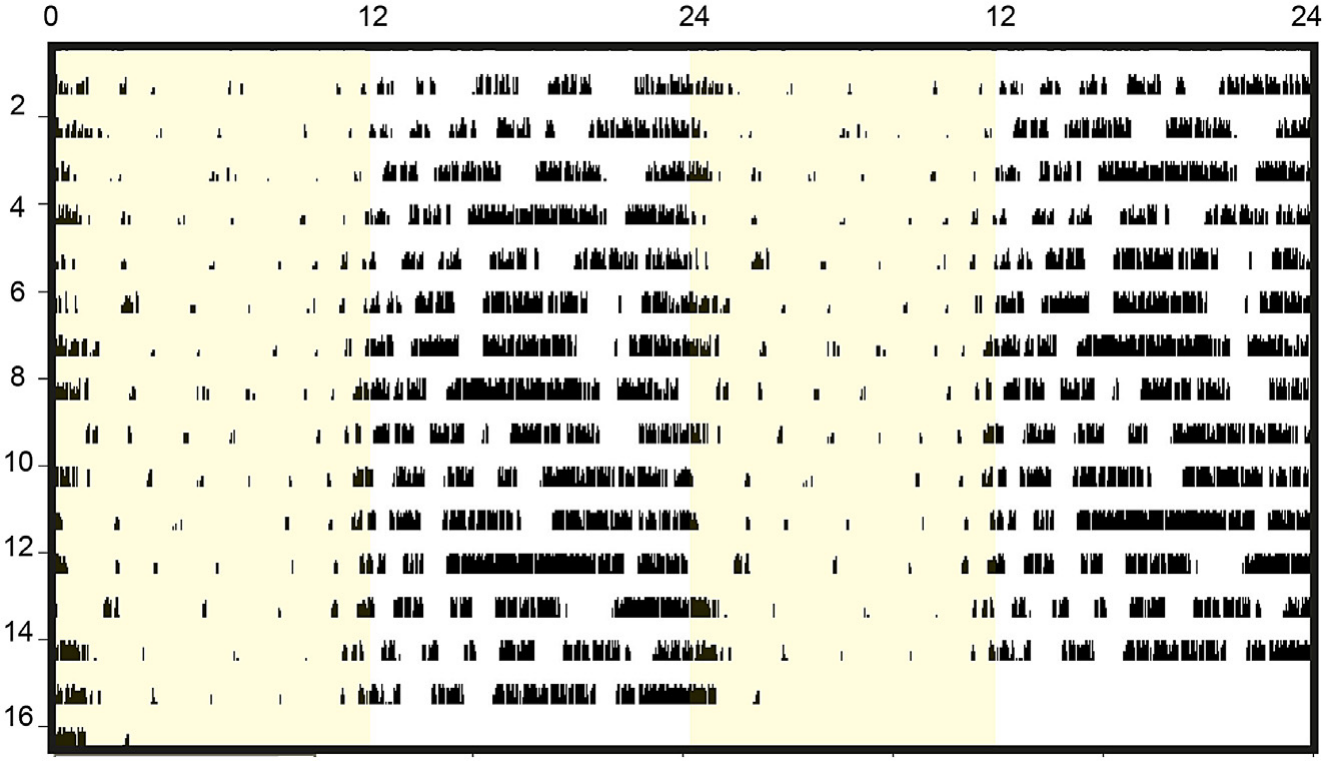
Circadian behavior analysis Double-plotted actogram showing wheel running activity for 2 weeks (y axis = number of days) in a 12:12 h light (yellow) /dark (white) cycle. Each horizontal line represents 48 h of activity and activity is represented by vertical bars.

***Note:*** While the data and protocol outlined here utilizes ClockLab software to analyze wheel running activity, any in-cage activity monitoring system which assesses activity in relation to the timing of the light cycle can be used. Mice are nocturnal animals and show maximum activity in the dark phase; ZT12-ZT24.

3. Analyze the locomotor activity (wheel-running activity) of the entrained mice for last 7–10 days (Figure 2). However, this step is not essential when working with a typical mice strain such as C57BL6/J, that can easily entrain to a LD cycle within one week.
  a. Use ClockLab Analysis 6 software (https://actimetrics.com/products/clocklab/)^3^ to inspect the behavioral rhythm as per manufacturer’s guidelines at 24 h period.

4. Discard animals that do not show robust rhythmic activity from the study.

**CRITICAL:** Use consistent analysis settings such as percentile/normalized actogram while assessing the circadian behavior using ClockLab.

## Key resources table

**Table:** 

REAGENT or RESOURCE	SOURCE	IDENTIFIER
**Chemicals, peptides, and recombinant proteins**		

RNAse*Zap*	Invitrogen	AM9780
Phosphate-buffered saline (10X) pH 7.4	Merck	P4474-1L
Diethyl pyrocarbonate (DEPC)	Sigma	D5758

**Critical commercial assays**		

RNeasy Micro Kit	Qiagen	Cat. No. 74004
Bioanalyzer RNA 6000 Pico Kit	Agilent	5067-1513
NEBNext® Ultra II Directional RNA Library Prep Kit	New England Biolabs	E7760L

**Experimental models: Organisms/strains**		

Mouse: C57Bl/6J background (8- 12 weeks-old) male or female	Wilcox et al.^4^	*Zfhx3*^Flox/Flox^;UBC-Cre^−^

**Software and algorithms**		

ClockLab Analysis Version 6	https://actimetrics.com/products/clocklab/clocklab-analysis-version-6/	Bunger et al.^3^
Galaxy	https://usegalaxy.eu/	Galaxy community
DryR	(https://github.com/naef-lab/dryR)	Weger et al.^5^

**Other**		

Aluminum foil	Any	N/A
Dissection tools (forceps, needles, razor blades, small scissors, big scissors, brush)	Any	N/A
Microscope glass slide	Thermo Scientific	12342108
Brain matrix	Kent Scientific	RBMS-200C
7000smz-2 Vibrotome	Campden Instruments	Model 7000smz-2
Adhesive tape	Any	N/A
Superglue	Any	N/A
RNase-free microfuge tubes	Invitrogen	AM12400

## Materials and equipment

**Table:** Equipment (Vibratome) settings

Frequency	70 Hz
Amplitude	1.00 mm
Section	250 μM
Advance	0.07 mm/s

• **0.1% DEPC treated water**: Add 1 mL of DEPC to 1 L of ddH20. Shake well to disperse the DEPC through the H2O under the fume hood. Incubate at 22°C–25°C for at least 12 h and autoclave at 15 psi on liquid cycle for 20 min to inactivate the remaining DEPC. Store at 22°C–25°C.
• **1X PBS (RNAse free)**: Add 100 mL PBS (10X) to 900mL DEPC treated water. Store at 22°C–25°C.
• **3% Agarose gel**: Dissolve 3 g in 100 mL PBS (1X, RNase free) solution. Allow it to set for 20 min at 22°C–25°C. Store at 4°C for 30 days.

***Note:*** The solutions are stable at 22°C–25°C, and there are no particular time constraints regarding their preparation or use.

**CRITICAL:** Diethyl pyrocarbonate (DEPC) is harmful with acute toxicity, Oral (Category 4), H302. Work under the fume hood and wash skin thoroughly after handling. Do not eat, drink or smoke when using this product.

## Step-by-step method details

### Brain dissection

**Timing: 2 days**

The brains are harvested from the entrained mice at designated time-points after cervical dislocation. Approximately 16 mice comprising of four biological replicates (3–4 animals per biological replicate) are used per time-point.

1. Following confirmation of stable entrainment, establish the correct dissection times for the time points needed.
2. Prior to dissection time, set up dissection area.
  a. Wipe down dissection area and dissection tools with RNAseZap.
  b. Cut aluminum foil into small squares (approximately 4 cm by 4 cm), one square for each animal. Place one foil square onto dry ice.
3. At the designated dissection time remove mouse from the cage, cull and dissect brain following institutional guidelines.

***Note:*** It is important to collect the tissue at a particular time-of-the-day even if it is not used for circadian studies as the transcript abundance greatly varies with time.

4. Place brain, cortex down, upon the foil square on dry ice.
  a. If regions of the brain other than the SCN are to be used in subsequent analysis, the orientation of the brain on the foil may need to be changed so that the region of interest is not in direct contact with the foil. See ‘troubleshooting’ section for details.
5. Allow the brain to freeze for 5 min. During this time, further dissections can be performed. (Figure 3).

**Figure 3:**
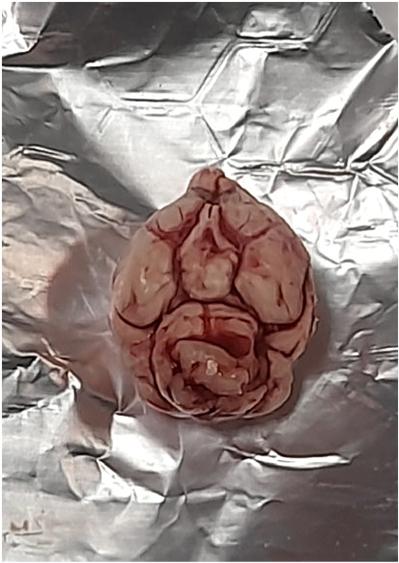
Dissected mouse brain on dry ice
6. Once frozen, store the brain at −80°C until ready to continue to SCN dissection as specified below.

**CRITICAL:** For dark-phase time points, perform the brain dissection under dim red light (no more than 50 lux). Desired intensity of the red light is maintained using filters blocking wavelengths below 600 nm. Avoid any exposure of light whilst collecting and freezing the brains.

### SCN collection

**Timing: 2 weeks**

The dissected brains are placed on dry ice and sliced in the coronal plane into 250 μM thick sections using a vibrating microtome. The brain slice between Bregma −0.3 mm and −0.8 mm,^6^ guided by the anterior anchor points with the intact optic chiasm, third ventricle and SCN is selected for dissection under chilled conditions.

7. Carry the stored mouse brains from −80°C freezer to the desired dissection area using a dry ice tray.
  a. Place brain matrix, sterile razor blades, forceps, dissection needles etc. on dry ice to chill.
8. Wipe the bench area, vibratome, light-dissection microscope etc. with RNaseZap to avoid contamination or degradation from RNAses.

**CRITICAL:** Wear gloves throughout the procedure and periodically change or wipe with RNAseZap.

9. Prepare the vibratome (https://campdeninstruments.com/products/7000smz-2-vibrotome) for coronal sectioning by following the specified guidelines in the equipment set up. Switch on the machine with the light source and scroll to the desired user and press [MENU]. The sectioning parameters associated with the user will display on the screen.
  a. Add ice to the tissue bath cooling jacket of the vibratome.
  b. Fill the metal tray with chilled 1% RNAse free PBS solution. See ‘troubleshooting’ section for details.
  c. Cover the vibratome metal chuck (removable tissue support) with tape. Use super glue to stick 1 × 1 cm block of 3% agarose gel at the curved edge of the chuck. This provides support for the mouse brain during gradual sectioning.
10. Separately, place the mouse brain (rostral to caudal) on the brain matrix and cut approximately 3 mm from the caudal end by razor blade to remove the cerebellum.

***Note:*** Ensure the tissue is perpendicular to the brain matrix and the cutting line is in parallel with the grooves in the brain matrix. In doing so, it keeps the left and right hemispheres aligned on the same coronal section in order to minimalize angular variation in the dissection plane. This is especially important for non-circular and small nuclei.

11. Apply a tiny drop of superglue on the metal chuck. Now, gently transfer the brain from the matrix to the metal chuck with the rostral end facing upwards and affix the chuck on the metal tray as specified in the instruction manual https://lafayetteinstrument.com/downloads/manuals/7000-v3.0%20Eng.pdf.

**CRITICAL:** The metal chuck should be submerged in the RNAse free PBS, covering the top of the brain specimen.

12. Manually set the bath to the start height following the manufacturer instructions.
13. Bring the cutting blade to a suitable position by setting the start position using [ADVANCE] and the rotary knob.
14. Press [SLICE ON/OFF] to begin and stop the sectioning.
  a. After a slice is cut, press [RETURN] to slide the blade back to the start position (Figure 5A).
  b. Section the mouse brain from rostral to caudal up to approximately 1.2 mm of Bregma.

***Note:*** Press [SLICE ON/OFF] to stop the sectioning once the blade reaches the top of the specimen, near the agarose block. After pressing [RETURN], if the slice is still attached to the specimen, dislodge it in the buffer gently with the help of a paint brush.

15. Gently transfer the mouse brain slice(s) with the help of a paintbrush onto the glass slide placed over a cold block.
16. Examine the mouse brain slice(s) collected between Bregma −0.3 mm and −0.8 mm for the presence of intact bilateral SCN (Figure 4) using the cell density contrast under light microscope. See ‘troubleshooting’ for more details.

**Figure 4:**
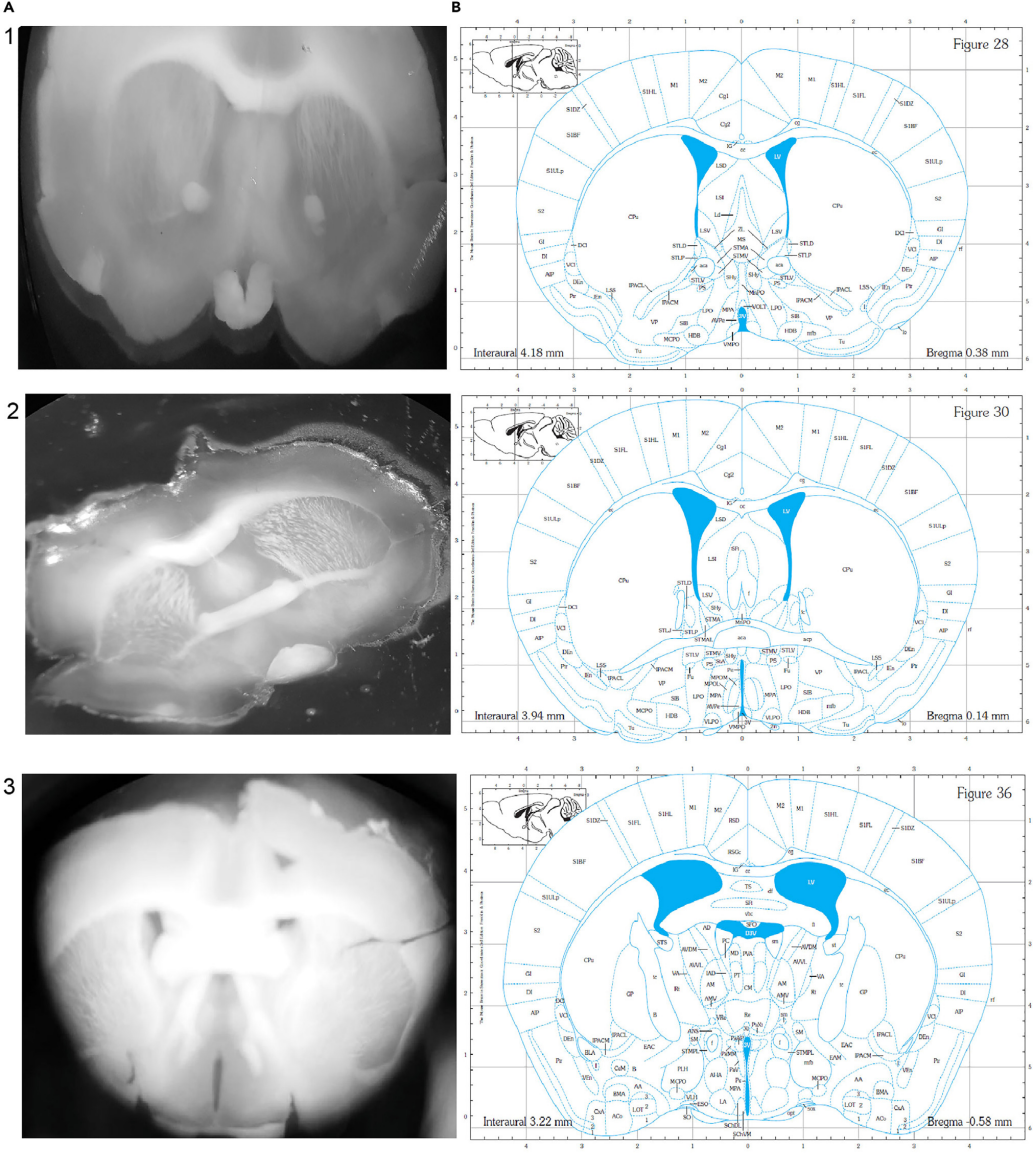
Reference Mouse brain slices Mouse brain slices (A) at 1) Bregma 0.3mm 2) Bregma 0.1 mm, slices prior to the SCN 3) Bregma -0.5 mm suitable for SCN sub-dissections. The corresponding Mouse Brain Atlas^6^ images are represented in (B) for reference.
17. Select the appropriate brain slice and tease apart the SCN from the surrounding hypothalamic tissue using pre-chilled dissection needles and forceps (Figure 5).

**Figure 5:**
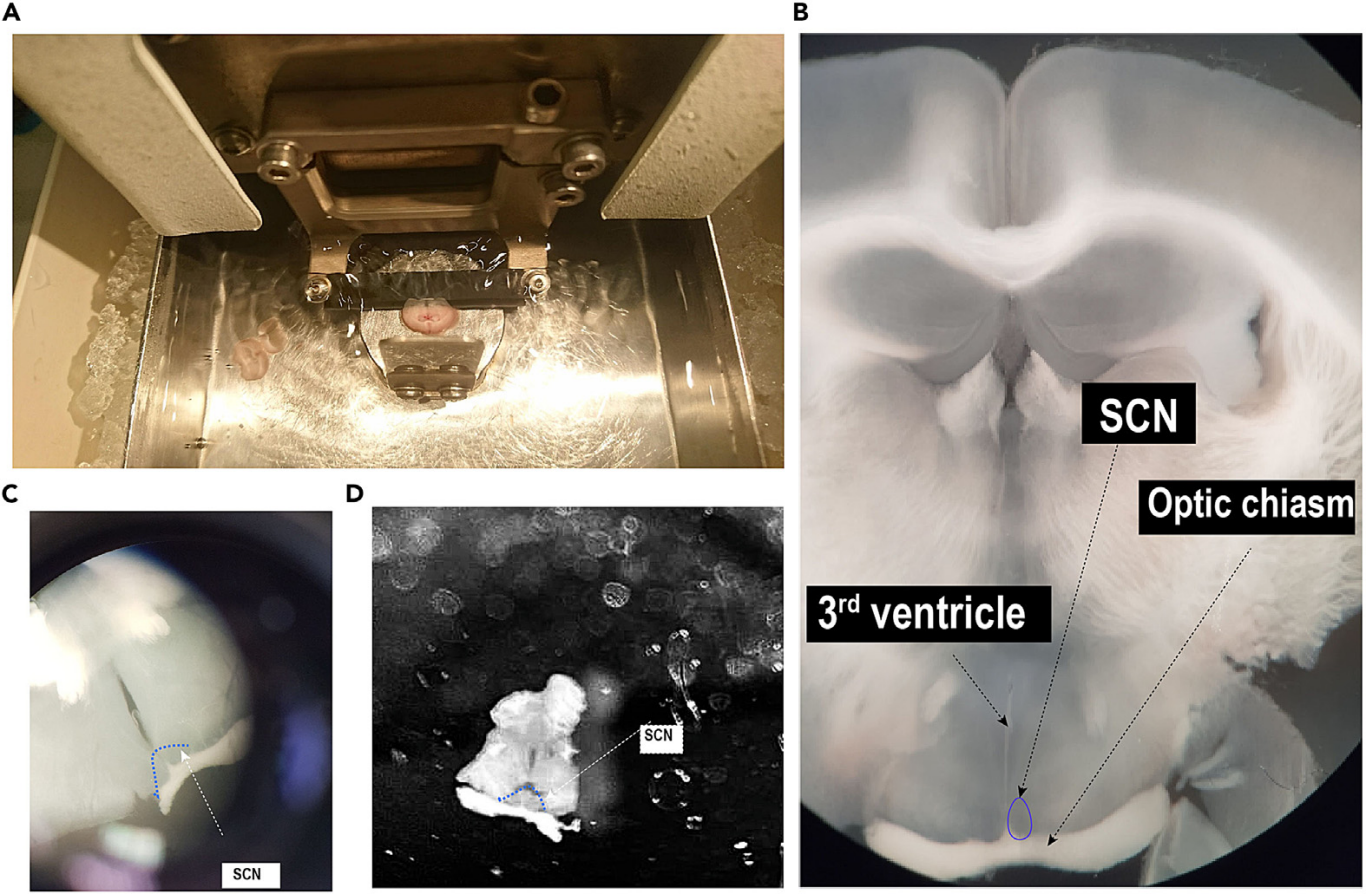
SCN sub-dissection (A) Mouse brain sectioning using vibratome under chilled conditions. (B) Microscope view of 250 μM mouse brain slice with the intact SCN. (C and D) Marked boundary of the SCN region (blue) dissected under microscope.
18. Aliquot 10 μL of Buffer RLT (RNeasy Micro Kit, supplemented with β-mercaptoethanol) on to the sub-dissected SCN and transfer into 1 mL RNAse free microfuge tubes (kept on ice) using 10 μL pipette tips.
19. Place the tube on dry ice.

**Pause point:** The dissected SCN can be stored in Buffer RLT for long term at −80°C.

### RNA extraction, library preparation and polyA enriched RNA sequencing

**Timing: 3 days**

The harvested SCN tissues are used for total RNA extraction to conduct polyA library preparation and sequencing. To increase the yield, SCN tissues collected at a single time-point are pooled prior to performing RNA extraction and purification. Post quality assessment, the extracted RNA is subjected to polyA library preparation and sequencing on Illumina platform.

20. Thaw the SCN tissues containing 1.5 mL tubes on ice for RNA extraction. Pool 3 individual SCN tissue samples suspended in Buffer RLT, collected at the same ZT in one separate RNAse free tube. This is referred as one biological replicate (BR) and labeled (for example; ZT2_BR1).

**CRITICAL:** The tissue samples should be thawed completely on ice before transferring to another tube. Special care should be taken while pooling the samples to avoid cross-contamination.

21. Adjust the sample volume to 350 μl with Buffer RLT and follow steps for RNA extraction using an RNeasy micro kit (Qiagen) as per the manufacturer’s instructions (https://www.qiagen.com/us/products/discovery-and-translational-research/dna-rna-purification/rna-purification/total-rna/rneasy-kits) for purification of total RNA from microdissected cryosections using laser microdissection systems. The extracted RNA is finally eluted in 14 μl elution buffer supplied with the kit. During the RNA extraction, perform on-column DNase digestion as per manufacturer’s guidelines.

***Note:*** Alternatively, if the brain tissue of interest is different (such as cortex, striatum etc.) any suitable commercial RNA extraction kit such as RNAqueous Kit (https://www.thermofisher.com/order/catalog/product/AM1912), PureLink RNA Mini kit (https://www.thermofisher.com/uk/en/home/life-science/dna-rna-purification-analysis/rna-extraction/rna-types/total-rna-extraction/purelink-rna-mini-kit.html) or TRIzol based RNA purification method^7^ can be adopted for RNA extraction.

**Pause point:** The extracted RNA can be stored for long term at −80°C.

**Figure 6:**
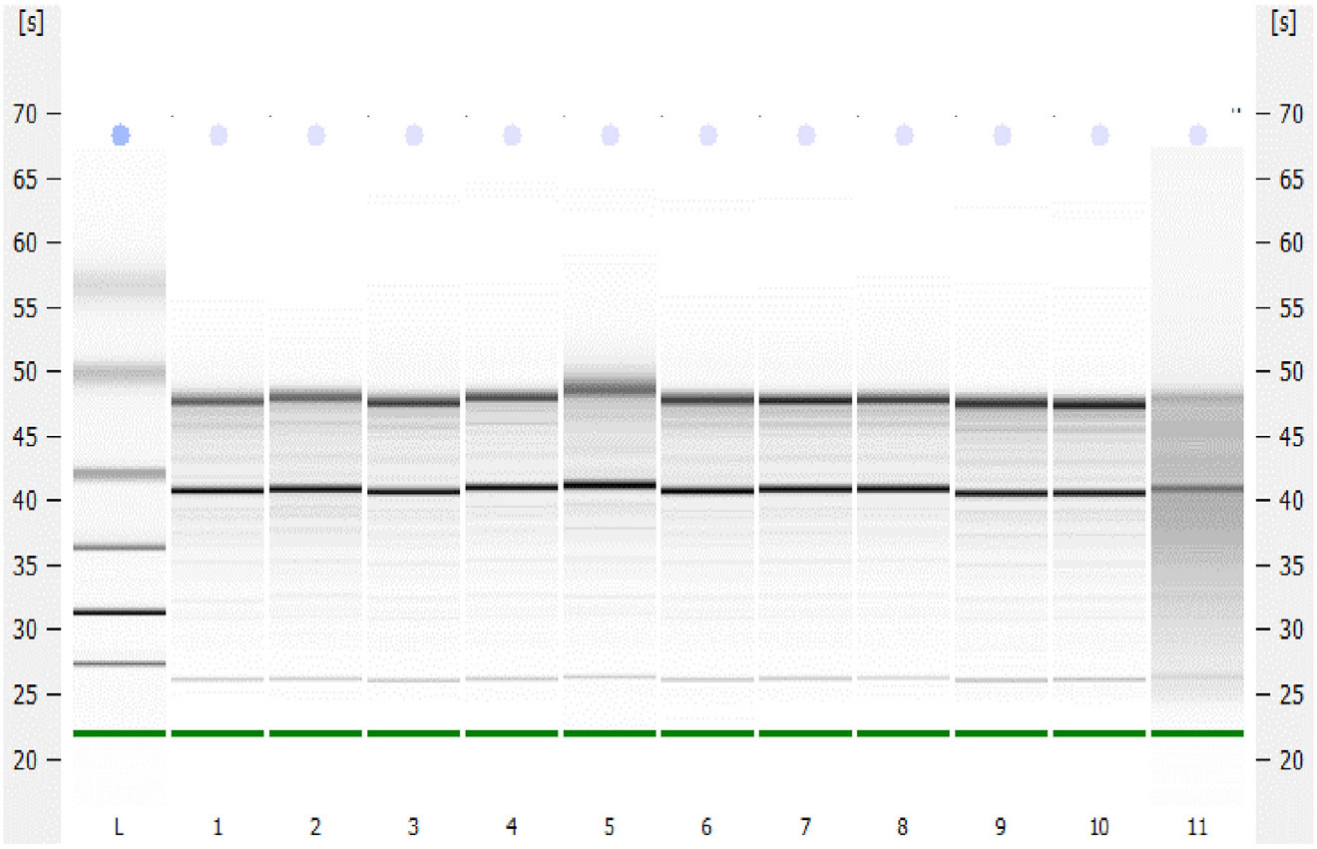
RNA quality assessment using Bioanalyzer Example of bioanalyzer report for quality assessment of the extracted RNA samples (lane numbered 1–11) along with the ladder (left, L). The two bands denote 28S (top) and 18S (bottom) rRNA.

***Note:*** Alternatively, RNA gel electrophoresis can be used to check for the integrity of the RNA, and Nanodrop to assess the purity and the concentration of the extracted RNA. Ideally, 260/280 ratio of ∼2.0 from Nanodrop is accepted as “pure” for RNA.

22. Inspect the quality and quantity of the extracted RNA for each sample on Bioanalyzer using Agilent RNA 6000 pico chip. For library preparation, do not include any RNA sample with RIN (RNA integrity number) < 7.0 (Figure 6). See ‘troubleshooting’ for more details.

23. Prepare libraries for sequencing, in accordance with the manufacturer’s guidelines for a NEBNext Ultra II Directional RNA Library Prep Kit (https://uk.neb.com/products/e7760-nebnext-ultra-ii-directional-rna-library-prep-kit-for-illumina#Product%20Information) and subsequently sequence the polyA enriched libraries on Illumina based platform (single or paired end).

## Expected outcomes

**Figure 7:**
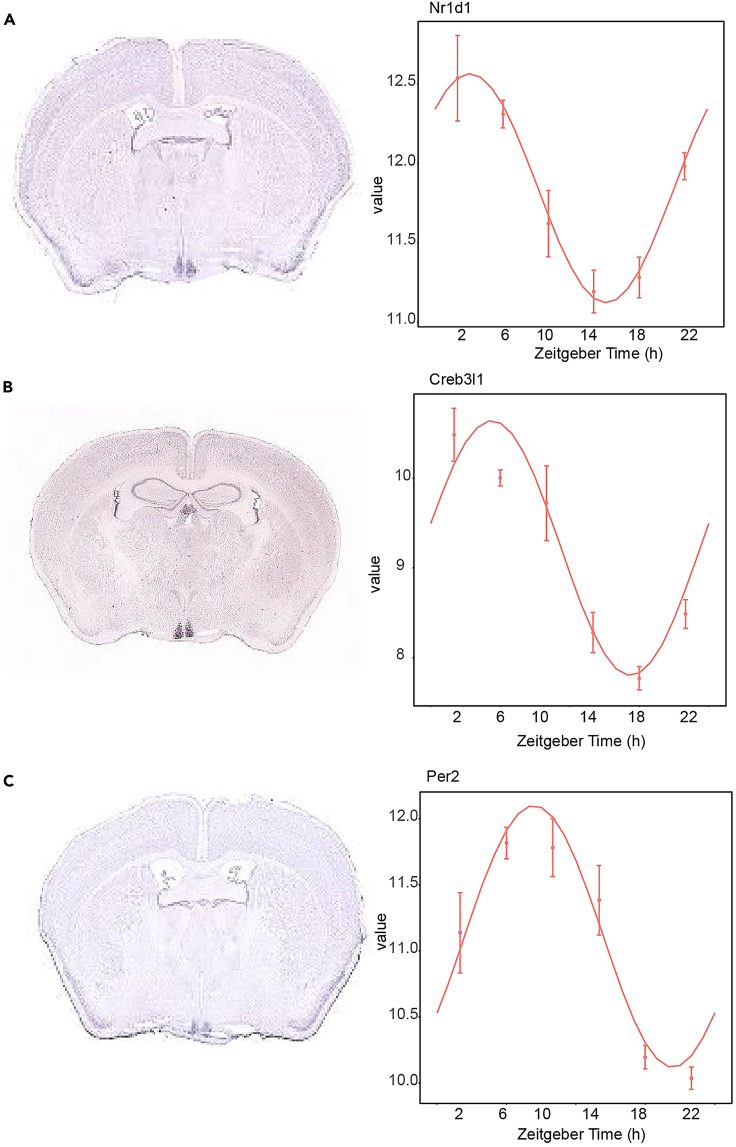
Rhythmic gene expression in the SCN (A–C) Representative examples of genes expressed in the SCN with in-situ hybridization images from Allen Brain Atlas (image credit: Allen brain atlas, Allen Institute^8^) (left panel) and gene transcription levels (logarithmic normalized counts) over 24 h (right panel) for (A) *Nr1d1*, (B) *Creb3l1,* (C) *Per2*. Data are represented as ± SEM.

The anatomically deep-seated SCN can be successfully harvested from the mouse brain using correct anchor points, optimal section settings and temperature control as described in this protocol. Slicing the frozen mouse brain tissue using ultra slow vibration speed (0.07 mm/s) eliminates the risk of accumulating uneven or broken sections and offers great advantage over cryostat sectioning when operated manually or at pre-programmed settings. In principle, this method of tissue dissection can easily be adopted to isolate any brain region of interest using cell density contrast. Further processing of the sub-dissected SCN tissues in RNAse free conditions will yield approximately 100 ng high quality RNA (RIN > 8.0) at a concentration of > 10 ng/ul. The extracted RNA from each sample when subjected to library preparation and sequencing would potentially result in > 40 million reads which is optimal to conduct diurnal transcriptome analysis to investigate cycling gene expression in the SCN as represented in Figure 7.

## Quantification and statistical analysis

**Timing: 2 days**

Here we provide steps to process the RNA-Seq data using Galaxy^9^ (https://usegalaxy.eu/) and identify transcripts with daily rhythmicity in the SCN using the R package dryR (Differential RhythmicitY analysis in R).^5^ To execute dryR, the user will be required to install R for the appropriate operating system at https://www.r-project.org/.

1. Assess the paired-end FASTQ files with FastQC (https://www.bioinformatics.babraham.ac.uk/projects/fastqc/) (cut-off Phred < 20) and remove Illumina adapters by TrimGalore (v0.4.3) (https://www.bioinformatics.babraham.ac.uk/projects/trim_galore/) .
2. Align the quality checked and trimmed reads to mm10 genome assembly using STAR^10^ (v2.7.8a) with MAPQ value for unique mappers set to 60. Use the binary alignment map (BAM) files to generate read counts per gene by FeatureCounts via Samtools (v1.11). Alternatively, FASTQ files can be uploaded on web application such as BioJupies (https://maayanlab.cloud/biojupies/) to generate raw count data.^11^
3. The gene counts generated in the above step are used to assess rhythmicity by R package dryR (Differential RhythmicitY analysis in R).^5^ dryR framework can analyze rhythmicity for one or more conditions. It can detect rhythmicity in a single condition (using the plot_single_cond() function) and compare rhythmicity across two or more conditions (using the dryseq() function). Alternatively, limma-voom method^12^ from the Bioconductor package-limma (v3.48.0) can be used to quantify differential gene expression, and the resulting normalized logarithmic CPM values can serve as an input for rhythmicity detection by applications such as ECHO,^13^ JTK_CYCLE^14^ etc.
4. Prepare the data matrix file from the raw count data (e.g., Table 1) to serve as an input file for dryR package available at https://github.com/naef-lab/dryR . In this instance, gene counts from four biological replicates per time-point (ZT2, 6, 10, 14, 18, 22) are used. Execute dryR specifying the condition(s) and time-series as instructed in the package guidelines. See ‘troubleshooting’ for more details.

**Table 1:** Example of count data as input to dryR

geneID	ZT2_1	ZT2_2	ZT2_3	ZT2_4	ZT6_1	ZT6_2	ZT6_3	ZT6_4	ZT22_1	ZT22_2	ZT22_3	ZT22_4
Sparcl1	133241	128950	135835	120630	121299	134442	134631	146724	153728	119037	141585	113176
Eef1a1	126918	157480	126393	180577	138867	145963	157508	144172	151068	136400	122563	126234
Scg2	103671	156188	86450	116774	91102	117539	121385	84991	95253	77344	96517	113110
Apoe	94421	156884	154180	108749	113970	97210	113397	146268	110930	83728	151492	92135
Hsp90ab1	93707	105659	88902	110551	82731	85625	94482	89968	96377	88660	73605	68876
Gprasp1	85157	106660	84077	94441	73734	94279	91610	86417	86648	85006	75326	76321
5. The output file will be .csv file specifying the corresponding amplitude (amp), phase etc. for each transcript (e.g., Table 2).

**Table 2:** Example of output file for one condition using the dryR function plot_single_cond?

geneID	pvalue	padj	Intercept	S1	C1	Phase	Amplitude
Per2	1.90E-14	1.20E-11	11.06178	0.803811	-0.57708	8.378389	2.273522
Dbp	1.76E-41	1.63E-37	11.62662	0.879305	-0.01367	6.059388	2.487051
Avp	2.61E-10	8.30E-08	12.6489	1.177705	-0.16611	6.535211	3.331052
Rgs16	4.37E-20	8.50E-17	13.02351	0.966868	0.612491	3.843105	2.734715
Cry1	2.06E-12	9.41E-10	10.87244	0.176704	-0.35751	10.24655	0.499794

***Note:*** The output file (Table 2) gives pvalue = p-value, padj = adjusted p-value, Intercept = mean, s1 = coefficients of the sine, c1 = coefficients of the cosine, phase = acrophase in hour(h), amp = log2 fold change (peak to trough) per gene/transcript.

## Limitations

Here we describe how to collect SCN during the day at distinct time-points, with a large number of biological replicates per time-point. Until now, the preferred method for collecting tissues like the SCN is either by tissue punch^15^ or laser capture microdissection (LCM).^16^ While the tissue-punch method often carries the risk of contamination from nearby tissue(s) and lack the desired precision, LCM is expensive and laborious especially when adopted for collection from large cohorts to achieve reasonable biological/technical replicates. For that reason, our protocol strikes a desired balance of collecting SCN tissues using low cost vibratome and dissection microscopes. Although this method is better over than the conventional tissue punch strategy, it still carries a minimal risk of contamination from nearby hypothalamic tissue.

In order to mitigate this risk, staining might help to locate specific brain regions, but the additional use of reagents and handling time has proven to be detrimental for high quality RNA extraction. Thus, this method of identifying SCN by cell density contrast between target and surrounding regions offer a huge benefit when performed cautiously. To maintain the integrity of the tissue, both sectioning and sub-dissection of the SCN from the mouse brain slice has to be performed side-by-side, not allowing for any pauses. This might result in lengthy processing time, but will not compromise the quality (and quantity) of the extracted genomic material. Thus, harvesting SCN for downstream RNA extraction for a large number of biological replicates, covering distinct times of the day, may require few weeks.

Bearing that in mind, this protocol describes the steps for transcriptome analysis at a resolution of 4 h during a day, and not for 2 days (48 h) or with higher resolution (2 h) to alleviate the risk of sample outliers and false-negatives in the rhythm detection. However, for significant rhythm detection from single circadian cycle, we chose a sampling interval of 4 h with increased number of biological replicates (n = 4) for each time-point, as per the sampling guidelines mentioned by Hughes et al. for genome-scale analysis of biological rhythms.^17^

## Troubleshooting

### Problem 1

Brain not properly frozen and foil wrapped (related to step 4).

### Potential solution

If the region to be dissected has come in direct contact with the foil, it will not be suitable for subsequent dissection. Discard the sample as per the institutional guidelines.

### Problem 2

The mouse brain sections are not even while vibratome sectioning (related to step 9).

### Potential solution

Make sure the 1% RNAse free PBS solution is chilled (0°C–4°C) before filling the metal tray. If the solution is not chilled, it will lead to thawing of the mouse brain which can result in uneven broken sections.

### Problem 3

SCN not visible under the dissection microscope (related to step 16).

### Potential solution

Adjust the reflective mirror at the base of the dissection microscope to light up the sample at different angles. Nuclei with denser cell bodies like the SCN will appear to be darker under direct reflected light, while its surrounding regions are lighter. Apart from the SCN it also works for other brain regions such as hippocampal formation, thalamic nuclei and zona inserta.

### Problem 4

RNA with poor integrity and quality (related to step 22).

### Potential solution

Inspect the integrity and purity of the extracted RNA. Proceed with the RNA extraction steps promptly, without any pause points, keeping the extracted RNA on ice to avoid RNA degradation.

### Problem 5

Unable to detect rhythmic gene expression using dryR (related to step 4 of quantification and statistical analysis).

### Potential solution

Use the correct function with the R package – dryR; dryseq_single for one condition and dryseq for multiple conditions specifying the conditions as [group] and time-points as [time].

## Resource availability

### Lead contact

Further information and requests for resources and reagents should be directed to and will be fulfilled by the lead contact, Patrick M. Nolan (pmnolan10@gmail.com).

### Materials availability

This study did not generate new unique reagents.

## Data Availability

The dataset supporting the current study have not been deposited in a public repository because of further experiments but are available from the corresponding author on request.
